# Honeysuckle extracts as a potential inhibitor of SARS-CoV-2 infection

**DOI:** 10.3389/fphar.2025.1517585

**Published:** 2025-04-16

**Authors:** Ping-Chang Lin, Ying-Ray Lee, Li-Teh Liu, Shyh-Shin Chiou, Po-Chih Chen, Ching-Yi Tsai, Jih-Jin Tsai

**Affiliations:** ^1^ Tropical Medicine Center, Kaohsiung Medical University Hospital, Kaohsiung, Taiwan; ^2^ Department of Microbiology and Immunology, School of Medicine, College of Medicine, Kaohsiung Medical University, Kaohsiung, Taiwan; ^3^ Master of Science Program in Tropical Medicine, College of Medicine, Kaohsiung Medical University, Kaohsiung, Taiwan; ^4^ Faculty of Post-Baccalaureate Medicine, College of Medicine, Kaohsiung Medical University, Kaohsiung, Taiwan; ^5^ Center for Tropical Medicine and Infectious Disease, Kaohsiung Medical University, Kaohsiung, Taiwan; ^6^ Department of Medical Laboratory Science and Biotechnology, College of Medical Technology, Chung Hwa University of Medical Technology, Tainan, Taiwan; ^7^ Taiwan Society of Thrombosis and Hemostasis (TSTH) Study Group, Taipei, Taiwan; ^8^ Graduate Institute of Clinical Medicine, College of Medicine, Kaohsiung Medical University, Kaohsiung, Taiwan; ^9^ Division of Pediatric Hematology and Oncology, Department of Pediatrics, Kaohsiung Medical University Hospital, Kaohsiung, Taiwan; ^10^ Center of Applied Genomics, Kaohsiung Medical University, Kaohsiung, Taiwan; ^11^ Department of Laboratory Medicine, Kaohsiung Medical University Hospital, Kaohsiung, Taiwan; ^12^ Department of Medical Laboratory Science and Biotechnology, Kaohsiung Medical University, Kaohsiung, Taiwan; ^13^ School of Medicine, College of Medicine, Kaohsiung Medical University, Kaohsiung, Taiwan; ^14^ Division of Infectious Diseases, Department of Internal Medicine, Kaohsiung Medical University Hospital, Kaohsiung, Taiwan

**Keywords:** honeysuckle, *Lonicera japonica* Thunb., SARS-CoV-2, COVID-19, extracts

## Abstract

**Background:**

In the current era of coronavirus disease 2019 (COVID-19), we were interested in searching for medications other than the currently available antiviral drugs Paxlovid and Molnupiravir that cause minimal side effects and do not harm the human body. Honeysuckle extract (HSE) is a traditional Chinese medicine (TCM) that has been shown to exert antiviral effects in other studies. However, no studies have indicated whether HSE has an inhibitory effect on SARS-CoV-2.

**Methods:**

We prepared HSEs from dried honeysuckle flowers. We performed a cell viability assay, median tissue culture infection dose (TCID_50_) assay, and qRT‒PCR, and calculated the virus titers using the Reed-Muench method to evaluate the inhibitory effects of aqueous and alcohol HSEs on SARS‒CoV‒2 and explore the possible underlying mechanisms.

**Results:**

In this study, post-treatment with HSE resulted in dose-dependent decreases in both the RNA levels and TCID_50_ of SARS-CoV-2 in Vero E6 cells; treatment with 50 μg/ml and 100 μg/ml alcohol HSEs achieved up to 95.323% and 92.587% inhibition, respectively. Moreover, pre-treatment with aqueous HSEs effectively reduced the RNA levels, and TCID_50_ of SARS-CoV-2 by up to 99.684%, and alcohol HSEs achieved up to 99.921% inhibition; both of these effects occurred in a dose-dependent manner.

**Conclusion:**

The results suggest that HSEs may have the potential to prevent SARS-CoV-2 infection.

## Introduction

Coronavirus disease 2019 (COVID-19) pandemic, which was caused by the emergence of severe acute respiratory syndrome coronavirus 2 (SARS-CoV-2), poses an unprecedented threat to public health and safety worldwide. The rapid spread of this virus and the profound impact of the pandemic on healthcare, economies, and societies (Lu et al., 2020; C; Wang et al., 2020). The emergence of the highly contagious and virulent SARS-CoV-2 virus in late 2019 quickly led to the global COVID-19 pandemic. This acute respiratory disease syndrome (ARDS), which is characterized by pneumonia, lymphopenia, and ARDS, is compounded by a hyperactive cytokine storm, resulting in a multifaceted health crisis with profound global impacts (Beeraka et al., 2021). COVID-19-induced ARDS is the primary cause of death among COVID-19 patients (Huang et al., 2020; Stebbing et al., 2021; Zhou et al., 2020). The fulminant cytokine storm has emerged as the predominant factor that causes death in these patients (Lee et al., 2021).

COVID-19 is a global health threat, and current treatments include Paxlovid (which is a SARS-CoV-2 protease inhibitor) (Marzi et al., 2022) and Molnupiravir (which interferes with the replication of SARS-CoV-2) (Kabinger et al., 2021); these are two major drugs that are used to treat SARS-CoV-2 infection, but some side effects, such as headache, emesis, loose stool, altered sense of taste, and bitter mouth, have been reported by the National Health Service England (NHS). Less commonly, muscular aches and elevated blood pressure have been reported in patients who were treated with Paxlovid (Hashemian et al., 2023); headaches and dizziness have been reported in patients who were treated with Molnupiravir (Singh et al., 2021); and diarrhea, nausea, and vomiting have been reported in patients who were treated with both drugs (Hashemian et al., 2023; Singh et al., 2021). Additionally, some cross reactions with other drugs occur; for example, Paxlovid is contraindicated with drugs that are primarily metabolized by CYP3A and for which elevated concentrations are associated with serious and/or life-threatening reactions (Hong et al., 2022). Regarding the safety of antiviral agents, the use of traditional Chinese medicine (TCM) warrants further exploration because various plant-derived metabolites in TCM have been shown to exert minimal toxic effects or to have only mild side effects (Poon et al., 2006).

**FIGURE 1:**
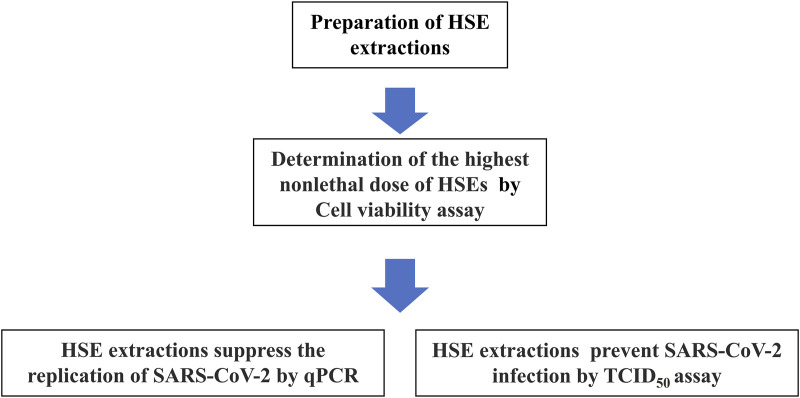
Steps to prepare and determine the highest non-lethal dose of HSEs in Vero E6 cells, as well as to assess the impact of different extraction methods on SARS-CoV-2 replication inhibition and reduction of infection potency, as outlined in a flowchart. The results indicate that pre-treatment yields better outcomes for both SARS-CoV-2 replication inhibition and reduction of infection potency.

Honeysuckle (*Lonicera japonica* Thunb.) is a TCM that is also used as a tea therapy (Mahboob et al., 2016; Rao and Al-Weshahy, 2008). Previous studies also revealed that honeysuckle-induced host innate miRNA expression may have the potential to block the spread and infection of various viruses. (Lee et al., 2017; Mahboob et al., 2016); additionally, it has been predicted that honeysuckle-induced miRNAs, including let-7a, are potential antiviral agents that protect against the SARS-CoV-2 virus and may suppress the replication of the SARS-CoV-2 virus at the miRNA level (Lee et al., 2021),but the effectiveness has not been verified with the actual virus. In this study, we performed *in vitro* experiments to investigate and confirm the antiviral effects of (HSEs) on a SARS-CoV-2 viral strain that was isolated from the nasopharyngeal swab of a patient. This confirms the therapeutic potential of HSEs against SARS-CoV-2. Additionally, at different treatment time points, alcohol HSEs can decrease the infectious titers of SARS-CoV-2 in both post-treatment and pre-treatment conditions. On the other hand, aqueous HSEs can inhibit SARS-CoV-2 replication and decrease the infectious titers of SARS-CoV-2 during pre-treatment, suggesting that HSEs have a significant impact on viral inhibition (Figure 1). Since HSEs are commonly used in dietary supplements, this provides valuable reference material for future research on the extraction methods and optimal treatment timing of HSEs against SARS-CoV-2.

## Materials and methods

### Preparation of HSEs

Dried honeysuckle flowers were purchased from Linyi Jin Tai Yao Ye Co. LTD in Shandong Province, China (voucher specimen number: 2011). The botanical identification of the honeysuckle (*Lonicera japonica* Thunb.) was confirmed by Dr. Guanghai Liu, a taxonomist at the Linyi Honeysuckle Institute in Shandong, China. To prepare a single dose of HSE, 10 g of dried flowers were extracted in either 500 ml of boiling double-distilled water or 500 ml of alcohol for 20 min before use (Lee et al., 2017). The metabolites fingerprint in the HSEs was analyzed by High Performance Liquid Chromatography (HPLC) (Supplementary Figures 1, 2). The major metabolites in the honeysuckle aqueous extracts and alcohol extracts were Chlorogenic acid and 3,5-Dicaffeoylquinic acid. The samples with Chlorogenic acid, Cynaroside and 3,5-Dicaffeoylquinic acid were used to be the standard and confirmed this analysis. Stock solutions of the extracts were prepared at a concentration of 100 mg/mL in pure water (aqueous extract) and dimethyl sulfoxide (DMSO, alcohol extract) and stored at −80°C for future use.

### Virus isolation

In the biosafety level 3 (BSL-3) laboratory, Vero E6 cells were seeded at a density of 1 × 10^5^ cells per well in a 24-well plate and incubated overnight to allow them to attach to the well. One hundred microliters of a swab-UTM (UTM Universal Transport Medium for Collection, Transport, and Preservation of Clinical Specimens for Viral Molecular Diagnostic Testing) sample with positive qRT‒PCR results was diluted by 2-fold in pretreated Dulbecco’s Modified Eagle Medium (DMEM) supplemented with 2× antibiotic-antimycotic (Thermo Fisher Scientific, United States) and added to the cell cultures. Then, the cells were incubated at 37°C in 5% CO_2_ for 1 h to allow virus attachment. Then, 600 μl of DMEM supplemented with 2% FBS was added to each well. After incubation, the cells were examined daily under a phase-contrast microscope to monitor the cytopathic effect (CPE). Once the CPE was observed, the presence of the SARS-CoV-2 virus in the culture supernatant was confirmed by qRT‒PCR. The clinically isolated virus was harvested at 3,000 rpm and 4°C and stored at −80 °C (Liu et al., 2022), and the success of virus cultivation was verified via qPCR (Liu et al., 2023).

### Cell culture, SARS-CoV-2 inoculation, and HSE treatment

In the BSL-2 laboratory, Vero E6 cells were used for SARS-CoV-2 propagation and routinely maintained in DMEM (Thermo Fisher Scientific, United States) supplemented with 10% fetal bovine serum (FBS), 1× antibiotic-antimycotic (Thermo Fisher Scientific, United States) and 1 mM sodium pyruvate (Thermo Fisher Scientific, United States) at 37°C in 5% CO_2_. “Post-treatment” was defined as follows: Vero E6 cells were first infected with the SARS-CoV-2 virus for 24 h and then treated with aqueous and alcohol HSEs. In contrast, “pre-treatment” was defined as follows: Vero E6 cells were first treated with aqueous and alcohol HSEs for 24 h and then infected with the SARS-CoV-2 virus.

### Cell viability assay

We used a Cell Counting Kit 8 (WST-8/CCK8) (ab228554, abcam, U.K.) to assess cell viability/proliferation in our study. Initially, 1 × 10^4^ Vero E6 cells per well were plated in 96-well plates and allowed to adhere overnight. Subsequently, the cells were washed with 1× phosphate-buffered saline (PBS) (Thermo Fisher Scientific, United States), and HSE was added to the cells, followed by incubation for the desired duration (e.g., 24 or 48 h) at 37°C in an incubator with 5% CO_2_. After another wash with PBS, we added 2.5 μl of WST-8 solution in 100 μl of culture medium to each well in the dark and then incubated the cells for an additional 1–4 h at 37°C. Finally, the increase in absorbance at 460 nm was measured (M. Wang et al., 2020).

### Virus titration/TCID_50_ assay

The SARS-CoV-2 virus named hCoV-19/Taiwan/KMUH-2/2020 (GASAID Accession ID: EPI_ISL_5395635) was isolated from UTM swabs. Vero E6 cells were maintained as stock cultures as described above, and 1 × 10^4^ cells per well were seeded in 96-well plates before infection for TCID_50_ assays. 100 μl of each solution and associated dilutions was inoculated onto each of 8 plate wells, so as to obtain 8 replicates per dilution (Baylis et al., 2008) The medium that was used for these dilutions was DMEM supplemented with 2% fetal bovine serum. After 3–7 days of incubation, the 96-well plates were fixed with 4% formaldehyde overnight and washed, and the remaining cells were stained with crystal violet (ASK RAPID GRAM STAIN, TONYAR Biotech. Inc., Taiwan.) for 2 h. After staining, the cells were thoroughly washed with distilled water and subsequently observed after drying. Then, the TCID_50_ value was calculated using the Reed-Muench method (Frilli et al., 2023; Lei et al., 2021).

### RNA extraction and qRT‒PCR

RNA was extracted from 140 μl of a swab-UTM sample using a QIAamp Virus RNA Mini Kit (QIAGEN, Germany) following the manufacturer’s instructions meticulously. qRT‒PCR was conducted using an Mx3000p qPCR system (Agilent, United States). *SARS-CoV-2* genomic RNA was measured by qRT‒PCR using SARS‒CoV‒2-specific primers and probes that were specific for the E, N, and RdRP genes. A 6-Carboxyfluorescein (FAM) fluorescent signal corresponding to any of the 3 genes was interpreted as a positive result (Corman et al., 2020). The primers that were used for the E, N, and RdRp genes were described in our previous study (Liu et al., 2021).

### Role of the funding source

The funders had no role in the study design, data collection or analysis, decision to publish, or preparation of the manuscript.

## Results

### The highest established nonlethal dose of HSEs in vero E6 cells

**FIGURE 2:**
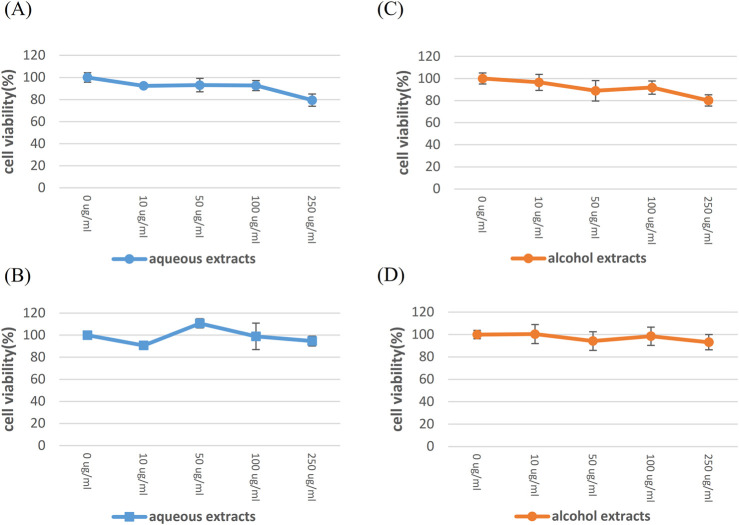
The highest established nonlethal dose of HSE in Vero E6 cells, for which SARS-CoV-2 was isolated. Vero E6 cells were exposed to various concentrations of HSE for 24 h and 48 h, aiming to determine the nonlethal dose. The cytotoxicity of these substances on Vero E6 cells was assessed using CCK-8 assays. The Y-axis of the graphs shows the mean % cell viability normalized to that in the control group (0 μg/ml; 100%). Each experiment was conducted in quadruplicate. Treatment with aqueous HSE for 24 h **(A)** and 48 h **(B)**, treatment with honeysuckle alcohol extracts for 24 h **(C)** and 48 h **(D)**.

To determine the highest nonlethal dose of HSEs that can be added to Vero E6 cells, we used the Cell Counting Kit 8 (WST-8/CCK8) to assess cell viability/proliferation. Aqueous and alcohol HSEs were added to Vero E6 cells, and the cells were incubated for 24 h and 48 h. Our experimental groups were treated with 0 μg/ml, 10 μg/ml, 50 μg/ml, 100 μg/ml, 250 μg/ml (Figure 2). After observing more than 90% cell viability in the presence of 100 μg/ml aqueous HSEs, we proceeded with the experiments using concentrations of 0 μg/ml, 50 μg/ml, and 100 μg/ml for both HSEs.

### The infectious titers of SARS-CoV-2 decreased after treatment with alcohol HSEs for 24 h

As previously mentioned, supernatants were collected at 24 h post-treatment with HSEs. These supernatants were subsequently subjected to analysis using TCID_50_ to determine the infectious titers (Table 1). At 24 h, the SARS-CoV-2 titers in the supernatants were reduced by 95.323% and 92.587% (after treatment with 50 and 100 μg/ml alcohol HSEs, respectively) compared to the titers after treatment with the vehicle (DMSO). The SARS-CoV-2 titers significantly decreased after treatment with the alcohol HSEs compared to those after treatment with the vehicle (DMSO), whereas no significant reduction was observed after treatment with the aqueous HSEs. (Data not shown) In summary, post-treatment with alcohol HSEs for 24 h resulted in a decrease in the infectious titers of SARS-CoV-2.

**TABLE 1:** Vero E6 cells were post-treatment with different doses of Alcohol HSE for 24 h

HSEs (μg/ml)		Result (TCID_50_/ml)	Reduction (TCID_50_/ml)	Inhibitory (%)
Alcohol HSE	0	10^8.33^	-	-
	50	10^7^	10^1.33^	95.323%
	100	10^7.2^	10^1.13^	92.587%

### The relative SARS-CoV-2 viral RNA levels decreased in a dose-dependent manner after 24 h of pre-treatment with aqueous HSEs

**FIGURE 3:**
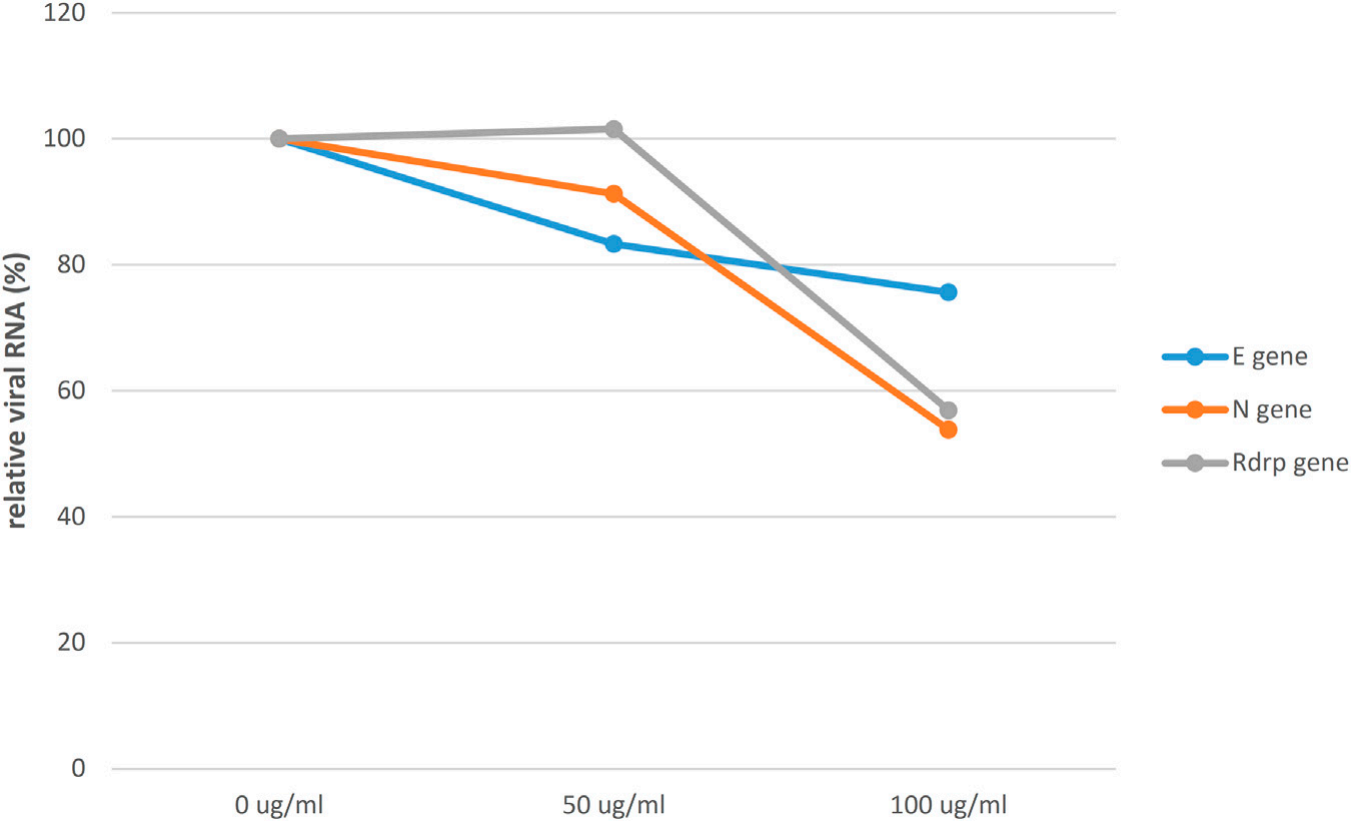
Relative SARS-CoV-2 RNA levels were decreased in a dose-dependent manner after a 24-h pre-treatment with aqueous HSE. Vero E6 cells were pretreated with HSE at concentrations of 0, 50, or 100 μg/ml for 24 h. Subsequently, the cells were washed with 1x PBS and infected with SARS-CoV-2 at a multiplicity of infection (MOI) of 0.1 for an additional 24 h. The viral yield in the cell supernatant was then quantified using qRT‒PCR and normalized to that in the control group (0 μg/ml; 100%). The Y-axis of the graphs represents the mean % relative viral RNA levels, which were normalized to that in the control group (0 μg/ml; 100%).

To evaluate the potential of HSEs to inhibit SARS-CoV-2 infection, Vero cells were pretreated with 0, 50, or 100 μg/ml HSE for 24 h. Subsequently, the cells were washed with 1× PBS and then infected with SARS-CoV-2 at a multiplicity of infection (MOI) of 0.1 for 24 h. The supernatants were harvested, and SARS‒CoV‒2 RNA levels were analyzed by qRT‒PCR to assess viral replication (Fig. 3). Notably, a dose-dependent decrease in viral replication was observed in cells that were pretreated with aqueous HSEs for 24 h. In summary, a dose-dependent decrease in the relative SARS-CoV-2 RNA levels was observed following a 24-h pre-treatment with aqueous HSEs.

### The infectious titers of SARS-CoV-2 were notably decreased after 24 h of pre-treatment with HSE

As mentioned above, cell supernatants were collected after a 24-h pre-treatment with HSE and subsequently subjected to TCID_50_ analysis to determine the infectious titers (Table 2). Compared with the titers in the supernatants of cells that were treated with the pure-water vehicle, the titers in the supernatants of cells that were pretreated with 50 μg/ml and 100 μg/ml aqueous HSEs were significantly decreased, with reductions of 96.838% and 99.684%, respectively. Similarly, pre-treatment with 50 μg/ml and 100 μg/ml alcohol HSEs led to substantial reductions in viral titers of 99.000% and 99.921%, respectively, compared to pre-treatment with the control (DMSO). In summary, there was a significant decrease in the infectious titers of SARS-CoV-2 after a 24-h pre-treatment with HSEs.

**TABLE 2:** Vero E6 cells were pre-treatment with different doses of HSEs for 24 h

HSEs (μg/ml)		Result (TCID_50_/ml)	Reduction (TCID_50_/ml)	Inhibitory (%)
Aqueous HSE	0	10^11^	-	-
	50	10^9.5^	10^1.5^	96.838%
	100	10^8.5^	10^2.5^	99.684%
Alcohol HSE	0	10^11.5^	-	-
	50	10^9.5^	10^2^	99.000%
	100	10^8.4^	10^3.1^	99.921%

## Discussion

### The side effects of existing COVID-19 treatments of COVID-19

Although the currently available medications, including Paxlovid and Molnupiravir, which are chemically synthesized drugs, show promise in the management of COVID-19, concerns remain regarding the potential side effects of these drugs.

A previous study demonstrated that honeysuckle plays a role in regulating the production of a wide range of cytokines, including IL-2, IL-7, and TNF-α (Huang et al., 2020).

Honeysuckle, which is scientifically known as *Lonicera japonica*, is a well-known medicinal plant that has a long history in TCM (Mahboob et al., 2016). The botanical significance of this plant not only attracts the attention contemporary researchers but also serves as a strong example of the extensive legacy of botanical drug healing practices. Honeysuckle is highly valued due to its multiple therapeutic properties, and it is prominently featured in medicinal teas because of its capacity to alleviate heat, purify the body, mitigate wind-heat ailments, and address a spectrum of conditions, including the common cold, fever, sore throat, heat induced toxin diarrhea, carbuncles, furuncles, chronic tonsillitis, and periodontitis. In addition to its role as a botanical drug remedy, honeysuckle is recognized for their potential cardiovascular benefits, including improvements in heart circulation, regulation of blood pressure, and reduction in cholesterol levels, and because it protects against coronary heart disease and angina pectoris (Golba et al., 2020; Wang et al., 2021).

Furthermore, honeysuckle has been a cornerstone of traditional medicine for centuries, being utilized both internally and externally, and it has been firmly established to have a good reputation for safety and efficacy across diverse populations. The enduring presence of this plant in traditional healing practices suggests that the prospect of utilizing HSEs could obviate the need for extensive safety testing in human subjects, thus presenting a compelling case for repurposing HSE as a pharmaceutical agent with a longstanding history of documented effectiveness and widespread acceptance in various cultural contexts (Shang et al., 2011).

### HSEs have the potential to combat SARS-CoV-2

Previous studies have revealed that honeysuckle-induced miRNAs, which include let-7a, have shown great potential in combatting SARS-CoV-2 infection. The mechanism of action requires the inhibition of viral replication at the level of complex miRNAs, thus providing new mechanisms by which to fight SARS-CoV-2 viruses (Lee et al., 2021).

In previous studies, a novel *in silico* bioinformatics workflow integrating multiple databases was developed to predict the potential use of honeysuckle (*Lonicera japonica*) as an anti-SARS-CoV-2 agent. The study utilized extracts from honeysuckle and Huangqi, which upregulate several critical microRNAs, including let-7a, miR-148b, and miR-146a, that are known to reduce SARS-CoV-2 pathogenesis. Additionally, these botanical drugs suppressed the production of proinflammatory cytokines, such as IL-6 and TNF-α, both of which have been implicated in the cytokine storm that is associated with ARDS, which is a major cause of death in patients with COVID-19. Furthermore, both botanical drugs partially inhibited the fusion of SARS-CoV-2 spike protein-transfected BHK-21 cells with the human lung cancer cell line Calu-3, which expresses ACE2 receptors. These findings suggest that these botanical drugs can inhibit SARS-CoV-2 Mpro activity, thereby mitigating viral entry and replication (Yeh et al., 2021).

### Previous studies lack validation for SARS-CoV-2

Previous studies focused on the potential of honeysuckle-induced miRNAs, including let-7a, miR-148b, and miR-146a, to suppress SARS-CoV-2 infection and to mitigate SARS-CoV-2 pathogenesis and suppress the production of proinflammatory cytokines such as IL-6 and TNF-α. These findings were validated using computational bioinformatics platforms and gene transfection techniques, but direct experiments using live SARS-CoV-2 were not performed (Lee et al., 2021; Yeh et al., 2021).

Given the rigorous regulatory framework governing SARS-CoV-2 research, coupled with the limited access to adequately equipped research facilities, conducting direct investigations into virus activity within conventional laboratories poses a substantial challenge. For instance, due to restrictions on the cultivation of the SARS-CoV-2 virus, which can only be performed in controlled BSL-3 or higher laboratories, the majority of laboratories are limited to using pseudovirus systems for experiments; such systems may lead to potential errors in experimental results.

### In our study, we use SARS-CoV-2 to validate the antiviral ability of HSEs

However, we have effectively overcome these obstacles by prioritizing comprehensive human training and allocating significant resources toward the establishment of cutting-edge, officially certified laboratories, thus enabling us to advance our understanding and research efforts in this critical area. In this study, we rigorously examined the antiviral effects of HSEs on clinically isolated SARS-CoV-2 virions, as opposed to pseudovirus models. This investigation was meticulously conducted in a highly controlled BSL-3 laboratory, where stringent adherence to the comprehensive laboratory biosafety protocols outlined by the Taiwan Centers for Disease Control (TCDC) was rigorously ensured throughout the entire research process.

In this study, our initial objective was to determine the highest nonlethal dose for Vero E6 cells, which are used for isolating SARS-CoV-2, in treatment with HSE. This step was crucial for preventing experimental errors due to cell mortality and ensuring the safety of the HSE.

Subsequently, both pre-treatment and post-treatment timepoints with HSEs were examined to assess changes in genes expression and infectious SARS-CoV-2 titers. In this study, the pre-treatment group was preexposed to a certain concentration of HSE, mimicking the scenario where cells are primed with a specific dose of HSE prior to SARS-CoV-2 virus infection. On the other hand, the post-treatment group was used to replicate the cellular state observed after administering HSE to cells that have already been infected. This approach aimed to elucidate the optimal timing for the administration of HSE as a therapeutic intervention for combating the SARS-CoV-2 virus. The investigation of both pre- and post-treatment conditions helps to provide insights into the potential efficacy of HSE and helps to identify the most favorable windows for its use in medicinal treatments.

In our investigation, when examining the cells subjected to HSE post-treatment, we observed a noteworthy reduction in the infectious titers of the SARS-CoV-2 virus within these cells. This finding suggests that HSE appears to exert a suppressive effect on the infectious capability of the SARS-CoV-2 virus, potentially influencing its ability to propagate within the cellular environment.

We found that compared with post-treatment, HSE pre-treatment effectively reduced the RNA levels and infectious titers of the SARS-CoV-2 virus, and pre-treatment was more effective than post-treatment. These findings suggested that pre-treatment with HSE can more effectively suppress the replication and infectivity of the SARS-CoV-2 virus.

In addition, we also tested aqueous and alcohol HSEs because, in addition to pure water extraction, alcohol extraction is a common method in TCM. In this study, alcohol HSEs showed better performance in reducing the infectivity of the SARS-CoV-2 virus (Miao et al., 2019; Wang et al., 2018; Zou et al., 2023).

In pharmacological research, aqueous and alcohol extracts may extract different metabolites, which could potentially affect antiviral efficacy. Therefore, we further utilized both aqueous and alcohol extracts for the study. At the post-treatment time point, only alcohol HSEs can reduce SARS-CoV-2 infection potency. However, at the pre-treatment time point, aqueous HSEs can inhibit SARS-CoV-2 replication, although its effect on SARS-CoV-2 infection potency reduction is weaker than that of alcohol HSEs. On average, alcohol HSEs demonstrate a stronger ability to reduce SARS-CoV-2 infection potency, but the ability of aqueous HSEs to inhibit SARS-CoV-2 replication is worth further investigation in future experiments.

### Limitations

There were certain limitations in our study. We demonstrated the inhibitory effect of HSE on the infection titers of SARS-CoV-2 *in vitro*. However, further research is warranted to investigate the efficacy of HSE in animal models and in human trials. Second, it should be noted that we utilized wild-type strains of SARS-CoV-2, which may differ from the currently prevalent variants of concern (VOCs), necessitating further exploration. Third, we were unable to determine whether the nontoxic doses that were used in this study corresponded to those used *in vivo*.

Importantly, the results are preliminary and need to be confirmed in further research. Further comprehensive investigations are needed to elucidate the complexities and specific mechanisms underlying the inhibitory effects of HSE on virus assembly or maturation, thereby contributing to a more thorough understanding of the potential applications of HSE in antiviral strategies.

## Conclusion

In this study, we maintained nonlethal concentrations of HSE on Vero E6 cells and utilized qPCR tests to determine the effectiveness of HSE in inhibiting SARS-CoV-2 replication. TCID_50_ tests were also conducted to assess the ability of HSEs to reduce virus infectivity, as determined by measuring infectious titers. Regarding the timing of the HSE administration to Vero E6 cells, pre-treatment refers to the pre-treatment of replicating cells with a specific concentration of HSE. This group models individuals who regularly incorporate honeysuckle into their dietary routine. Following pre-treatment, the SARS-CoV-2 virus was introduced to evaluate the impact of HSE pre-treatment on infection. Conversely, post-treatment endeavors to replicate cellular conditions after the administration of HSE as a medicinal intervention. This group models treatment with HSE after SARS-CoV-2 infection occurs.

By selecting the optimal time point for HSE treatment, we were able to discern its optimum efficacy in inhibiting the replication and infectivity of the SARS-CoV-2 virus. As a result, we not only confirmed the therapeutic potential of HSE against the SARS-CoV-2 virus but also conclusively determined that incorporating honeysuckle as a dietary supplement represents a better approach for potentially preventing SARS-CoV-2 infection.

## Data Availability

The raw data supporting the conclusions of this article will be made available by the authors, without undue reservation.
