# Supplementary material for: Honeysuckle extracts as a potential inhibitor of SARS-CoV-2 infection
Source: Front Pharmacol. 2025 Apr 16;16:1517585. doi: 10.3389/fphar.2025.1517585 (PMC12083240; doi:10.3389/fphar.2025.1517585)
Supplement: Supplementary file 1 [file DataSheet1.docx]

**Supporting Materials**

**Supplementary Fig. 1 The fingerprint of honeysuckle aqueous extracts compared with standard (STD)**

**Supplementary Fig. 2 The fingerprint of honeysuckle alcohol extracts compared with standard (STD)**

**
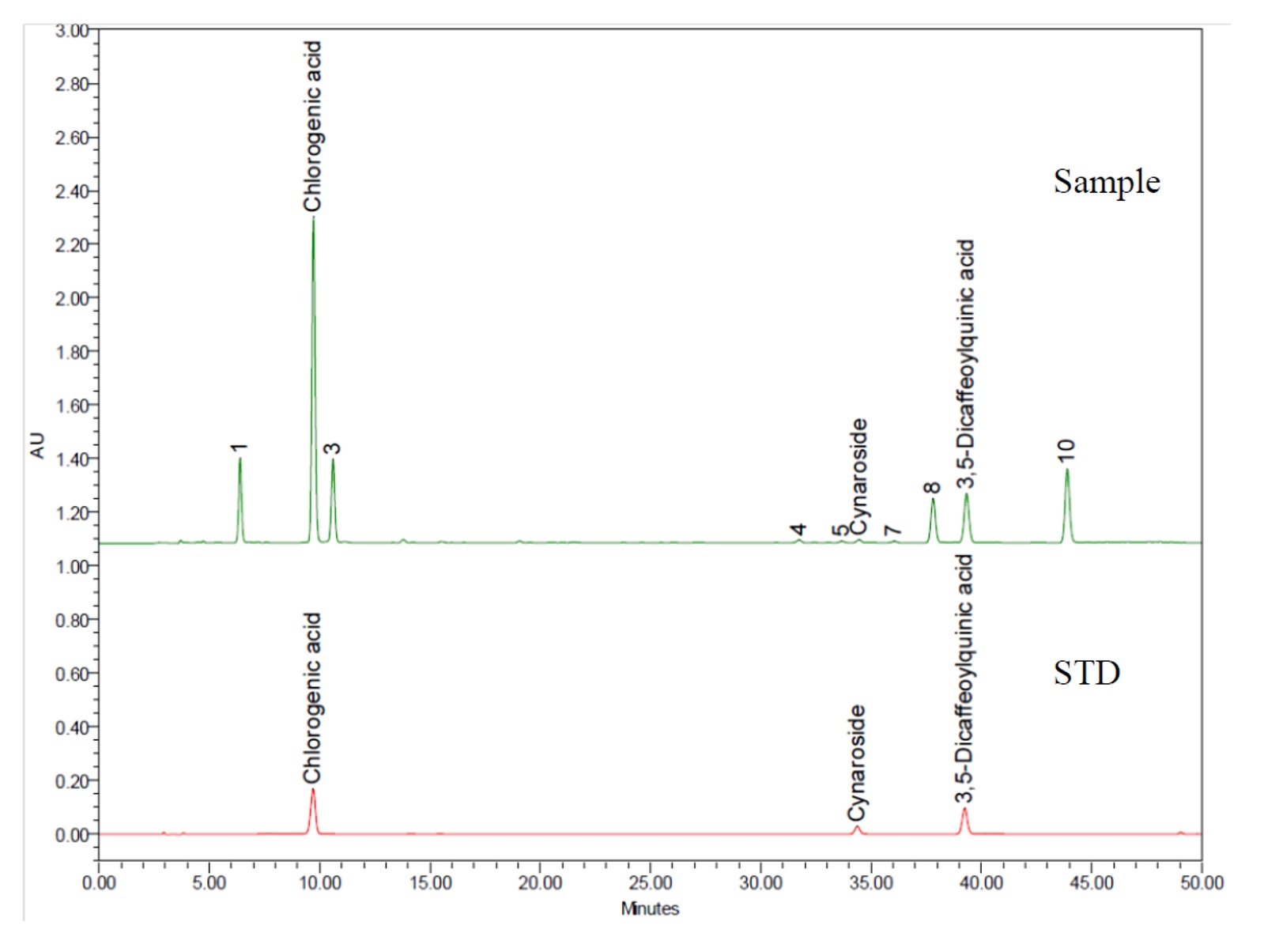
Supplementary Fig. 1 The fingerprint of honeysuckle aqueous extracts compared with standard (STD)**


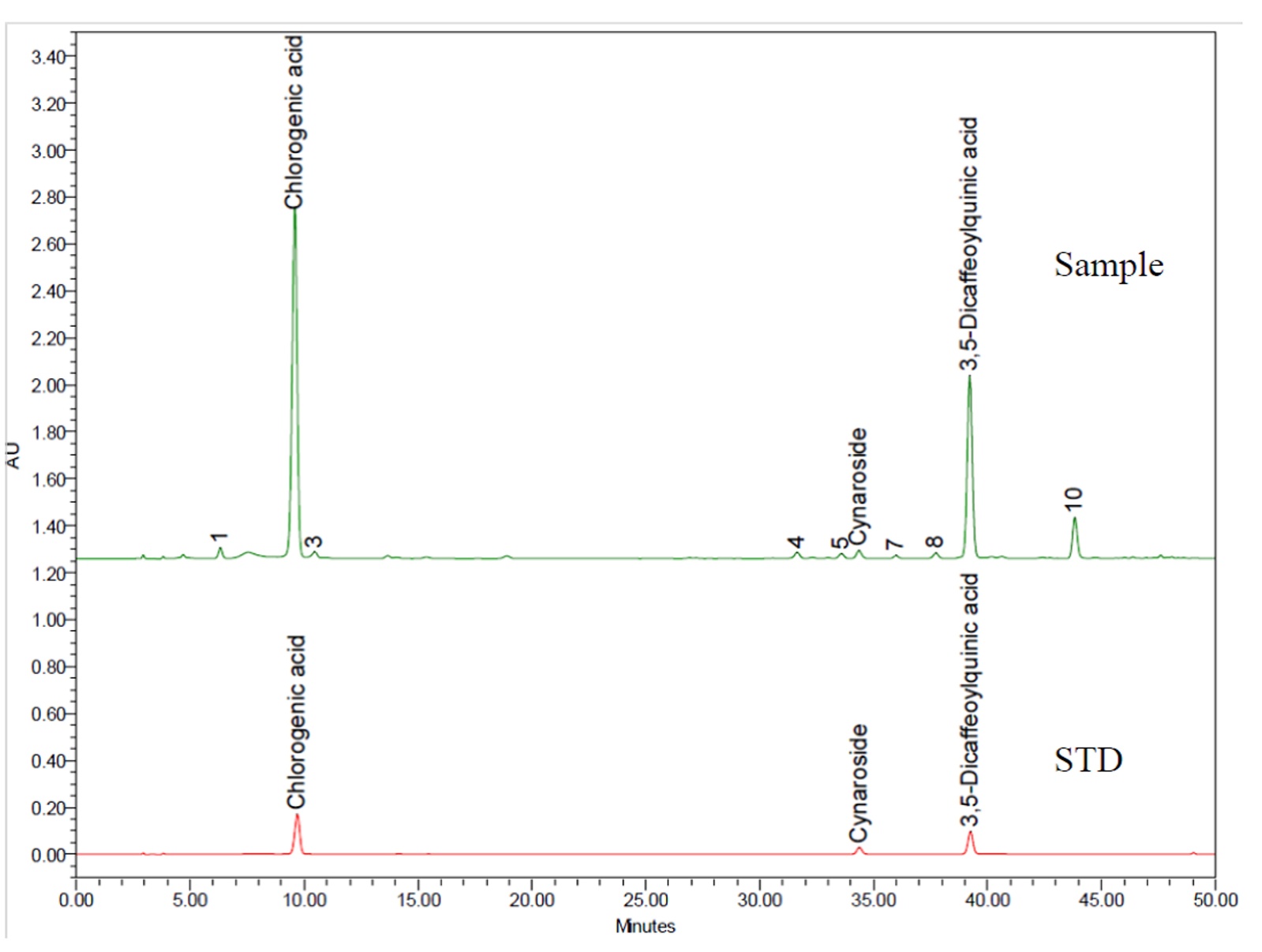


**Supplementary Fig. 2 The fingerprint of honeysuckle alcohol extracts compared with standard (STD)**
